# South American Archaeological Isotopic Database, a regional-scale multi-isotope data compendium for research

**DOI:** 10.1038/s41597-024-03148-9

**Published:** 2024-04-04

**Authors:** Luis Pezo-Lanfranco, Patricia Mut, Juan Chávez, Thiago Fossile, André Carlo Colonese, Ricardo Fernandes

**Affiliations:** 1 Institute of Environmental Science and Technology (ICTA), Universitat Autònoma de Barcelona, Cerdanyola del Vallès, Spain; 2 Department of Prehistory, Universitat Autònoma de Barcelona, Cerdanyola del Vallès, Spain; 3 Departamento de Antropología Biológica, Universidad de la República, Montevideo, Uruguay; 4 Department of Anthropology, University of California, Riverside, USA; 5 Observatorio de Patrimonio Cultural y Arqueológico – Instituto de Investigaciones Antropológicas y Arqueológicas, Universidad Mayor de San Andrés, La Paz, Bolivia; 6 Department of Archaeology, Max Planck Institute of Geoanthropology, Jena, Germany; 7 Faculty of Archaeology, University of Warsaw, Warsaw, Poland; 8 Arne Faculty of Arts, Masaryk University, Brno, Czech Republic; 9 Climate Change and History Research Initiative, Princeton University, Princeton, USA

**Keywords:** Biogeochemistry, Biochemistry

## Abstract

The South American Archaeological Isotopic Database (SAAID) is a comprehensive open-access resource that aggregates all available bioarchaeological stable and radiogenic isotope measurements, encompassing data from human individuals, animals, and plants across South America. Resulting from a collaborative effort of scholars who work with stable isotopes in this region, SAAID contains 53,781 isotopic measurements across 24,507 entries from individuals/specimens spanning over 12,000 years. SAAID includes valuable contextual information on archaeological samples and respective sites, such as chronology, geographical region, biome, and spatial coordinates, biological details like estimated sex and age for human individuals, and taxonomic description for fauna and flora. SAAID is hosted at the PACHAMAMA community within the Pandora data platform and the CORA repository to facilitate easy access. Because of its rich data structure, SAAID is particularly well-suited for conducting spatiotemporal meta-analyses. It serves as a valuable tool for addressing a variety of research topics, including the spread, adoption, and consumption intensification of food items, paleo-environmental reconstruction, as well as the exploration of mobility patterns across extensive geographic regions.

## Background & Summary

### The south american archaeological isotopic database (SAAID)

The South American Archaeological Isotopic Database (SAAID)^1^ is an open-access data resource that compiles available stable (*δ*^13^C, *δ*^15^N, *δ*^34^S, *δ*^18^O) and radiogenic (^87^Sr/^86^Sr) isotope measurements reported for human, animal, and plant bioarchaeological remains from South America. For data collection, a systematic review was carried out of available scientific publications (i.e., peer-review indexed journals) and grey literature.

SAAID^1^ is hosted within the PACHAMAMA data community at the Pandora data platform (https://pandoradata.earth/dataset/saaid_v-2-0_2023), and is a partner member of the IsoMemo network initiative of autonomous isotopic databases (https://www.isomemo.com). SAAID^1^ is also stored at CORA (*Catalan Deposit of Research Data -* 10.34810/data602).

### Stable isotope studies in south america: brief background

**Fig. 1:**
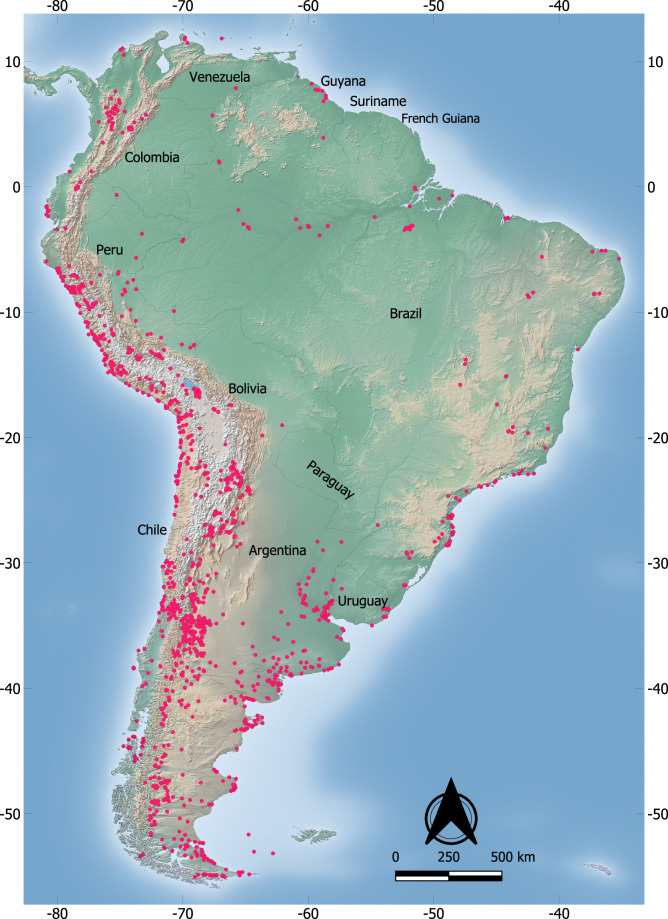
Spatial distribution of archaeological sites with isotope data included in SAAID. Map generated in QGIS v.3.2.8 (https://www.qgis.org) with a raster basemap from Natural Earth (https://www.naturalearthdata.com/).

Stable isotope analysis is one of the fastest-growing scientific methods to study past human societies and is often used to infer dietary, mobility, and environmental information from archaeological materials^2–4^. Stable carbon and nitrogen isotopes, for example, have been used for over 45 years^5,6^ as their first use in South America dates back to the 1980s^7–10^. Extensive chronological and geographic scales have been covered ever since^11–24^, especially since the 2000’s, but considerable gaps remain (Fig. 1).

In South America, stable isotope data collection for archaeological research is uneven across countries and regions, and less intense than in Europe or North America. The relative delay of around 20 years in the popularization of isotopic methods in South America could be explained by the limited diffusion of the research advantages of employing stable isotope analyses in archaeology, the lack of local facilities and funding for sampling and analyses, issues with sample preservation (e.g. poorly preserved collagen), the relatively high cost of isotopic analyses in laboratories abroad, and the scarce number of scholars who specialized in the subject. The reasons that influenced differences in the adoption of isotope analyses were also likely structural. Between the 1970s and 2000s several south American countries were under military dictatorship (e.g. Brazil, Argentina, Chile, Peru, Uruguay) or subject to armed internal conflicts (e.g. Colombia, Peru) with severe impacts on sociopolitical life, academic systems, funding institutions, and technological development^25,26^.

Currently, there is renewed interest in using stable isotope analyses in archaeological research in South America, especially on bone and tooth bulk collagen, and bioapatite. This growing trend could be the result of the popularization of stable isotope analyses worldwide, increased access to commercial and academic facilities, and improvement in laboratory protocols for maximizing and optimizing sampling extraction and pre-treatment^27–31^. Equally important, countries such as Argentina, Brazil, and Chile have invested in training (e.g., National Council for Scientific and Technological Development - CNPq, Brazil) and laboratory facilities (e.g., São Paulo Research Foundation - FAPESP, Brazil; Consejo Nacional de Investigaciones Científicas y Técnicas - CONICET, Argentina), in collaboration with external partners. These actions have increased access and subsequent application of stable isotope studies in domestic archaeological research in recent decades^32–34^.

In a bibliometric assessment conducted through 2023, there are a total of 569 archaeological reports based on stable isotopes, with a disproportionate data production in countries such as Argentina (201 publications), Peru (163 publications), Chile (80 publications), and Brazil (50 publications). The number of publications in all remaining countries does not exceed 75 titles, even considering unpublished dissertations and grey literature. No data were found for Paraguay and Suriname.

Except for Argentina, where a few local laboratories have focused on isotopes in archaeological research and allowed for expressive and uninterrupted scientific production in the field since the second half or the 1980s^32^, most countries’ data production is rarely local. In Peru, for instance, due to a lack of stable isotope laboratories, scientific production is largely dominated by researchers and scientific laboratories from North America and Europe, who import materials under scientific collaboration agreements^34^. The same trend is observed in Ecuador, Colombia, Venezuela, Guyana, and Bolivia. In Chile and Brazil, studies are mostly dominated by local scholars working with non-local laboratories, while in Uruguay, isotopic research is entirely local, although of low scale.

Isotopic have been employed to address a variety of historical, paleo-environmental, and paleo-ecological questions in the region^32–34^. Whereas ecological studies are well supported by extensive research databases^35–37^, there are no centralized storage repositories that facilitate access to isotopic data from South American bioarchaeological samples. As a result, a relatively large amount of data potentially valuable to address large-scale and long-term archaeological questions is scattered, unorganized, and often inaccessible for the rest of the world.

To promote isotope-driven archaeological research in South America, we present here SAAID^1^, an open-access database that collects stable (*δ*^13^C, *δ*^15^N, *δ*^34^S, *δ*^18^O) and radiogenic (^87^Sr/^86^Sr) isotope measurements from human, faunal, and plant remains retrieved from South American archaeological contexts. SAAID^1^ brings together data published over the last four decades in a wide range of sources (i.e., archaeological, palaeoecological, bioarchaeological, and forensic reports). These sources included: i) scientific publications in peer-review journals; ii) unpublished academic dissertations and theses; iii) official research reports; iv) edited books and monographs available in local repositories (e.g. universities, and research institutions); v) printed reports or regional journals that, depending on the year of publication, may not be available online; and, lastly, vi) unpublished raw data provided by researchers via personal communication.

SAAID^1^ adheres to the fundamental FAIR principles^38^ and addresses the increasing need for data consolidation within archaeological science and the wider trend towards data democratization and sharing. Its extensive temporal and spatial coverage make it a a valuable resource for conducting meta-analyses at various scales. This includes the study of subsistence systems, patterns of human and animal mobility, paleo-environments and human-induced ecological changes across diverse regions in South America^2,3,32,33^. SAAID^1^ can also be employed to identify research gaps for future field works and isotopic analyses. The database is open to continuous data flow and will be subject to future updates as new data is released.

## Methods

### Data compilation and building strategies

SAAID^1^ was built in five operative phases (OP) through Systematic Bibliography Review (SBR), which is an approach particularly useful to integrate all relevant data from several independent studies on a certain topic. SBR follows an explicit and systematic search method guided by a well-defined theme question and clear inclusion criteria^39^.

#### OP 1: Systematic Review of Isotopic Data

The criterion for the inclusion of isotopic data in SAAID^1^ was the presence of isotopes of archaeological relevance in the publication’s content. After establishing inclusion and selection criteria and keywords, researchers received training to ensure uniform criteria application, then proceeded to source references for inclusion in the dataset. Data collection was limited to bioarchaeological samples located within South America, with no temporal restrictions. Isotopic data were retrieved from primary sources (scientific articles, book chapters, archaeological reports, and academic dissertations) and secondary sources (academic reports containing various archaeological data and previous compilations or databases), regardless of the language, with a predominant focus on English, Spanish, and Portuguese, and particular attention to languages used by scientific teams that have worked in South America (e.g., the Japanese, the German, and the Polish archaeological missions in Peru, the French mission in Brazil etc.).

No exclusion criteria were applied to human, animal, and plant isotope values from archaeological origin or modern specimens obtained from archaeological studies if they were used to build isoscapes of food-webs. Isotope data of modern species registered in ecological databases were excluded. Amino acid isotopic data, less common isotopic proxies (e.g., Zn, Mg) or isotopic measurements from soil, sediments, and water samples were excluded from the current version of SAAID^1^ but may be added in future versions. Isotope values, including those from older publications that may not meet current quality standards, were compiled.

The search for isotopic data consisted of the following approaches: a) searching for relevant bibliographic references using online search engines and keyword combinations by modern country; b) identifying additional data sources (e.g., cross-references, grey literature, references not available online) from the references listed in collected publications; and c) requesting raw data from libraries that possess old and rare printed books or directly from authors.

We gather the sources from seven electronic databases widely known globally (Google Scholar, PubMed, Web of Science, Scopus) and regionally (SciELO, Redalyc, Dialnet), and other databases from national scientific agencies (CAPES-Brazil, Conicet-Argentina, Conicyt-Chile, Concytec-Peru), in that order. To locate data, we employed the following keywords: ‘isotopes’, ‘carbon’, ‘nitrogen’, ‘sulphur’, ‘dietary reconstruction’, ‘diet’, ‘mobility’, ‘archaeology’, ‘palaeoecology’ + ‘[name of country]’, and combinations.

#### OP 2: Selecting suitable sources for data compilation

This phase included data examination and scrutiny within the chosen publications. The identification and preliminary selection of studies that could provide data was done by evaluating the titles and abstracts identified through the search terms. One screening was conducted, observing the defined criteria for inclusion and exclusion, and reading the paper in its entirety. This procedure began with a review of the most recent bibliography to detect possible data repetitions. To be considered eligible, we assessed the entire text to confirm the presence of isotope data. The ultimate selection of publications for inclusion in the dataset hinged on the identification of isotope values in tables, appendices, and/or supplementary materials within the source. In certain cases, we garnered details pertaining to the nature and context of sites including their locations and chronology, from sources distinct from those providing isotopic data. In others, we reached out to request unpublished raw data or to supplement data that had not been adequately published.

#### OP 3: Extracting and organizing data

Data extraction was carried out on a per-country basis using pre-structured Microsoft Excel spreadsheets. The extracted data encompassed isotope values of archaeological significance, δ^13^C, δ^15^N, δ^34^S, δ^18^O, and ^87^Sr/^86^Sr, ^84^Sr/^86^Sr and lead isotopic ratios. These isotope values were derived from human and animal tissues, such as bone and tooth bulk collagen (sequential dentin collagen), bone bioapatite and tooth enamel, hair keratin or fur sections, and mummified or raw tissues (e.g., muscle, skin, tendon etc.). Isotope values from archaeological plant samples and from modern plants of archaeological importance, which were employed as baseline references in archaeological studies, were also included. The extraction began with the more recent publication and concluded with the older one. If the same data was reported across multiple publications the most recent publication was selected as the most reliable source of data and metadata. Data compilation was conducted between December 2021 and July 2023.

#### OP 4: Data curation

Following the data compilation and organization of databases by country (‘national databases’), a meticulous site-by-site review was conducted to identify potential data entry errors. This phase also involved cross-referencing individual data with supplementary contextual information and ensuring data compatibility across partner databases within the IsoMemo network.

#### OP 5: Data aggregation in SAAID

A definite version of SAAID^1^, revised in format and content was created at this final operative phase. National data was combined into the single SAAID^1^ database. At this time, the dataset went through a third revision, looking for copy and formatting errors, and then the collected data were tested and validated through exploratory meta-analyses using the R-based Data Search and Spatiotemporal Modelling 23.10.1 software available from Pandora & IsoMemo software platform (https://pandoraapp.earth/app/iso-memo-app)^40–42^. The dataset was edited in Excel and then converted in OpenDocument and CSV formats, ready to manage and export to statistical software. Finally, SAAID^1^ was uploaded to the final repositories.

## Data Records

SAAID^1^ is hosted in CORA (*Catalan Deposit of Research Data*) platform and Pandora platform into the repository of *PACHAMAMA: Archaeological Databases from South America* Community. The structure of SAAID^1^ is based on the structure of other similar databases and previous compilations of bioarchaeological isotopic data^43–46^. SAAID^1^ is made available as a single Excel spreadsheet file with three separate sheets for data: (1) humans, (2) fauna, and (3) plants, and one for metadata description (SAAID_V.2.0_2023.xlxs), and derived versions in open-access formats (a ODS file - SAAID_V.2.0_2023.ods; and three CSV files - SAAID_V.2.0_2023_Humans.csv; SAAID_V.2.0_2023_Animals.csv; SAAID_V.2.0_2023_Plants.csv). An additional text file (Readme.txt) contain the technical information of the database and a brief description of the methods and content. Whereas all these files are available in the Pandora platform, CORA platform^1^ hosts only the basic files (.ods and.txt).

While the three spreadsheets follow a common structural framework, variations exist in certain fields among humans (76 fields), fauna (75 fields), and plants (57 fields) due to the distinct characteristics of data collected for each sample type. The first row is the only one with descriptors (field names). The metadata sheet within Excel and ODS files provides a description of each field.

Each row or entry contains individual isotopic measurements plus additional contextual data. The current version of SAAID^1^ compiles 53,781 isotopic measurements in 24,507 entries divided into three general categories: humans (n = 15,385; 62.8%), fauna (n = 5,508; 22.5%), and plants (n = 3,611; 14.7%). These data originate from 2,139 archaeological and modern sites.

Each entry has a unique identifier (sequential integer number), often representing a single individual/specimen. However, there are cases where a single individual may have multiple entries. For instance, humans with two or more isotope values from hair segments or dentin collagen sections are reported across multiple rows. Additionally, users may find two or more rows for cases where more than one sample from the same individual was analyzed or if the same sample was analyzed multiple times (repetitions/replication). In such cases, a text note is included in the *Extra information* or *Comments* fields.

The different dataset fields are organized into seven primary thematic sections:

### Entry identification

The includes the modern-day name of the country in which the archaeological site is located, the name of the archaeological site from which the data are derived, and the archaeological ID of the human, animal, or plant specimen, as given in the original source. Country names are organized alphabetically, and site names are arranged chronologically. The ID of individuals and specimens usually follows the same order as reported in the source.

### Archaeological site data

The section provides geographical and environmental data such as the regional location of the site, biome classification, geographical coordinates in decimal degrees (WGS84), altitude in meters above the sea level, and site type and function when available. SAAID^1^ provides two classifications for the biome: a more generic classification based on Olson (2001)^47^, updated by WWF (2006)^48^ and CIESIN (2012)^49^, and a more specific classification used in each country, or a more detailed ecological classification based on Griffit *et al*.^50^ (see http://ecologicalregions.info/htm/sa_eco.htm). The exact location of the site was recorded whenever possible, if not, the site was located and georeferenced using Google Maps^®^ and Google Earth^®^, and reported with an approximate radius of uncertainty in km. Only a few sites could not be localized. The classification of the site by type and function is based on archaeological references. This section was challenging to build due to the lack of coordinates provided by publications and the absence of reliable GIS for archaeological sites in most countries, with exception of Brazil (https://sicg.iphan.gov.br/), Peru (https://sigda.cultura.gob.pe/#), and Colombia (https://geoparques.icanh.gov.co/#/sitiosatlas/).

### Chronology

This section reports both absolute (uncalibrated and calibrated radiocarbon dates) and relative (estimated period according to regional or national classifications) chronological data. Chronology was standardized on a regional and country basis, enabling comprehensive comparisons across various geographic and sociocultural contexts. The chronology for each entry is classified as relative or absolute. The individual/specimens’ chronology is attributed following a hierarchical approach using the highest dating resolution available: (1) direct individual/specimen dating (direct dating of bones or teeth); (2) burial dating (dating of associated materials); (3) site dating (general dating for archaeological site); (4) dating of archaeological culture (according to a wider periodization). SAAID^1^ includes numeric values for the date range associated to each individual/specimen (in BCE/CE), along with the corresponding archaeological or cultural period and/or phase, when available. Negative values are used to report BCE dates, and positive values for CE dates. For direct individual/specimen dating, SAAID^1^ reports the Dating Method (such as 14 C, 14C-AMS, Thermoluminescence etc.), Lab Code, uncalibrated date (date BP and their standard deviation), whenever available. Calibrated dates, when available, were reported in three different systems typically used by archaeologists: BCE/CE, cal BP, and BP at 2 sigma, as they appear in the source. The material subject to dating, from the sample or associated with the sample, is also reported. If the material was not reported the cell is empty.

### Individual’s data

For humans, this section includes sex and age estimates, including age cohort and wide age-ranges, whenever available. Sex and age, estimated through osteological analysis, were collected from the original or associated bibliographic sources. For humans, SAAID^1^ reports the individual ID, biological sex, the wide category of age-at-death (e.g., Infant-2, Child-1, Middle Adult etc.), and corresponding age-range in years (e.g., 1–5 years; 6–8 years, 35–50 years etc.)^51^. If the original source reports an age-range for individuals it was included in the dataset as published. When the authors do not set specific age-ranges but age-categories, we provide a corresponding approximate age-range (see Metadata spreadsheet). For animals and plants, the taxon, species, and other relevant features such as associated environment (e.g., terrestrial fauna, fresh-water fauna, terrestrial flora) and tissue description of the sample (e.g., wool, fur, seeds) are provided. For consistency, it was necessary to standardize bone and tooth names (in this case, changing FDI Notation or others to a standardized Anthropological Notation), and simplifying excessive description or redundant information, such as the number of hand or foot phalanges (for instance, “1^st^ right phalange” to “phalange”) or long bones side, to improve further comparisons. While acknowledging the presence of certain limitations, significant efforts were invested in standardizing bioarchaeological markers, such as age and sex from the multiple notations of the primary sources to a unique and standardized scale^51^. This standardization has resulted in a unified scale that facilitates broader comparisons.

### Isotopic values

Isotopic measurements are reported according to material. The first subsection reports carbon, nitrogen, and sulfur isotope values from bone and dentin collagen of humans and fauna, or from bulk tissues in plants. The second subsection includes carbon, oxygen, strontium, and lead isotope values from bioapatite. The dataset also lists isotopic values from hair keratin, muscle, and skin for humans, and from wool, fur, flesh, and other tissues for fauna. Isotopic values from coprolites or waste (guano) were included with plant data. SAAID^1^ reports δ^18^O_C_ and δ^18^O_P_ results for oxygen isotopic ratios according to the standard reported in the original publication, relative to VPDB (Vienna Pee Dee Belemnite) or VSMOW (Vienna Standard Mean Ocean Water).

SAAID^1^ also provides information on “Tissue-Age,” which refers to the age category of the tissue or segment sampled, in years. This information is useful for comparing individuals by age, which can help assess intracommunity dietary differences and migration patterns. SAAID^1^ provides an approximate and reliable estimate of tissue age for most evaluated tissues. However, estimating tissue age for bones can be challenging due to differences in bone turnover rates that depend on an individual’s age and the type of bone sampled, varying between 1 to 10 years (or more) before death^52–54,55^. To overcome this limitation, tissue-age is classified as the same age-at-death category for subadults, assuming that tissue formation occurred relatively close to death. For adults of any category, tissue-age is labeled as “Adult” due to the less reliable estimation of true tissue-age.

In the case of teeth, SAAID^1^ adopts the chronology suggested by The London Atlas of Human Tooth Development and Eruption^56^, which provides an approximate age-range of formation for each tooth or section and have shown a good global performance^57^ (see Supplementary Information). Although other dental development and eruption charts could be more suitable for Amerindian populations^58^, they do not have easy application for assigning age to dental sections or segments. Caution is recommended for the use of these proposed tissue-ages since the dental development of Andean populations may differ significantly (considerably precocious) from other Amerindians and populations around the world^59,60^. Finally, skin, muscle, and hair represent the last few months before death, based on previous studies^61,62^.

### Extra information and comments

Includes additional contextual data, comments and extra references, that may be useful for understanding the data source. The *Additional Info Source* field provides key references that contain supporting data, such as maps, site descriptions, bioarchaeological data, and other relevant information about individuals and samples. The *Comments* field contains observations about the nature of the study or any other pertinent information.

### References

The final section gives the bibliographic reference of the primary source (*Reference*) or previous compilations (*Compilation*) using the APA citation style. The author(s) name together with year of publication are also given in a separate field. Aiming reliability, and whenever available, SAAID^1^ lists digital object identifiers (DOIs), URIs, or URLs plus direct links to data repositories (*Link to source*). If an online repository is not available, as in the case of old printed books, only the reference was included.

## Technical Validation

Upon completion of the selection and extraction stages, samples were classified according to the availability of standard preservation criteria. This classification, which considers preservation markers in bioapatite (e.g., crystallinity indexes^30^) and collagen (e.g., collagen yield, atomic C:N atomic ratio, Wt%C, Wt%N, Wt%S^27,28^), is recorded in the *Preservation criteria* field as follows: 1 = complete preservation markers; 2 = just C:N atomic ratio; and 3 = no preservation markers). Older publications tend to not report these criteria. In early papers, authors mention the usage of quality indicators but do not necessarily report them.

Samples with preservation criteria following outside standardly accepted ranges are included in the dataset, so that this data can be used in studies of sample preservation. In such cases, SAAID^1^ users are advised to filter out these values if they require only top-quality data.

For some original sources, isotopic values were only reported as population means or are included only in scatter plots. We contacted publication authors whenever possible to obtain raw data; if this was not possible, we recorded the mean values, number of individuals and standard deviation. In a few exceptional instances, we were able to recover values from scatter plots using the software WebPlotDigitizer v.6.4^63^. Additionally, we included other relevant information, such as the type of measurement (i.e., IRMS or AMS) reported for carbon stable isotope ratios. This distinction is crucial since carbon values measured using AMS may not be reliable^64,65^. This supplementary information was documented in the *Comments* field of the database.

## Usage Notes

### SAAID limitations

SAAID^1^, however, comes with several limitations that are important to consider. One significant limitation of the data compilation is the absence of precise absolute chronology across many sites, potentially impacting the accuracy of temporal studies. Radiocarbon data was often poorly reported in older publications in South America (e.g. missing lab codes, sample description – marine, freshwater, terrestrial, organic, or inorganic –, no explanation if the date is reported as conventional or calibrated etc.). Different from specialized radiocarbon datasets which reports dates from sites and different materials^66,67^, SAAID^1^ only reports dates related to the entries (individuals/specimens) and generic periodization.

The distribution of samples and measurements across countries and regions is uneven, which can lead to regional imbalances in data representation and limit the potential for wide-scale spatial comparisons. Asymmetries in the number of individuals by sex and age are common among the reported sites. Lastly, oxygen and strontium data should also be expanded with contextual data from the landscape and terrain because SAAID^1^ does not include isotope values from meteoric and superficial waters or sediments that might be useful for spatial mobility studies^43,68^.

Finally, most original sources normally report a map with site location, but do not report precise geographical coordinates. Despite having valuable references compiling sites’ coordinates^66,67,69^, there may be disagreements in the reported coordinates for the same site between two or more references. Whenever possible, we obtained coordinates from official or government repositories. If these were not available, we report sites’ locations using Google Earth coordinates because they provide more reliable visual approximation to the sites. However, it is important to note that Google Earth employs the *WGS84/Pseudo-Mercator* projection, making it impossible to accurately estimate areas and positional precision.

### Updating SAAID

SAAID^1^ is a dynamic source and allow amendments and new data additions. New data will be included in the database as it is released, with a periodicity of approximately 12 months (once a year) in both repositories. Following FAIR principles, the new versions of SAAID will be overwritten and made available under a unique DOI. Updated and older versions will be accessible in current repositories conveniently labeled to avoid confusions. We encourage independent researchers to contribute new data through the Pandora repository. Any future data contributions will be integrated into the original SAAID^1^ file using the recommended format and assigned a DOI for easy identification. The data will then undergo editing and validation by project curators.

### Disclaimer

This article was peer reviewed in 2023 based on SAAID_V.2.0_2023, available on the CORA platform at the time^1^. This version compiles isotopic data from a total of 676 sources, including complementary archaeological literature^70–717^.

### Supplementary information

Supplementary Information

## Data Availability

The statistical analysis and modeling employed for the database validation was performed with Pandora & IsoMemo Applications. Source code for spatiotemporal model (TimeR) Data Search and Spatiotemporal Modelling 23.12.0.2software – Time R Model is available online (https://pandoraapp.earth/app/iso-memo-app), and also available for download at GitHub (https://rdrr.io/github/Pandora-IsoMemo/iso-app/).
