# Supplementary material for: South American Archaeological Isotopic Database, a regional-scale multi-isotope data compendium for research
Source: Sci Data. 2024 Apr 4;11:336. doi: 10.1038/s41597-024-03148-9 (PMC10995213; doi:10.1038/s41597-024-03148-9)
Supplement: Supplementary file 1 — Supplementary Information [file 41597_2024_3148_MOESM1_ESM.pdf]

Supplementary Information

Dental-age tables used by SAAID

Based on The London Atlas of Human Tooth Development and Eruption

AlQahtani, S. J., Hector, M. P., & Liversidge, H. M. Brief communication: the London atlas of human tooth development and eruption. *Am. J. Phys. Anthropol.* **142**, 481–490 (2010).

AlQahtani, S. J., Hector, M. P., & Liversidge, H. M. Accuracy of Dental Age Estimation Charts: Schour and Massler, Ubelaker, and the London Atlas. *Am. J. Phys. Anthropol.* **154**, 70–78 (2014).

Deciduous dentition

Maxilla	Cusp initiation	Crown ½	Crown complete	Root ½	Root complete	Apex closed
di1	-0.4	-0.6	0.1	0.9	1.5	2.5
di2	-0.5	-0.8	0.4	1.5	1.8	2.5
dc	-0.8	0.4	0.7	2.0	2.5	3.5
dm1	-0.4	0.4	0.6	1.5	2.3	3.5
dm2	-0.7	0.4	0.7	1.8	2.5	3.5

Mandible	Cusp initiation	Crown ½	Crown complete	Root ½	Root complete	Apex closed
di1	-0.4	-0.6	0.0	0.6	2.0	2.5
di2	-0.5	-0.7	0.1	0.9	2.0	2.5
dc	-0.7	0.4	0.9	1.5	2.5	3.5
dm1	-0.4	0.3	0.6	1.5	2.5	3.5
dm2	-0.9	0.5	0.9	2.0	3.0	3.5

Permanent dentition

Maxilla	Cusp initiation	Crown ½	Crown complete	Root ½	Root complete	Apex closed
I1	0.4	1.2	4.5	7.0	8.5	11.0
I2	0.8	2.5	5.0	7.5	9.0	11.5
C	0.7	2.5	5.0	9.5	12.5	14.0
Pm1 (P3)	2.5	4.5	6.0	10.5	12.5	15.0
Pm2 (P4)	3.5	5.5	6.5	11.0	13.0	14.0
M1	0.4	2.2	3.5	6.5	8.5	9.5
M2	2.5	6.0	8.0	10.5	14.5	16.5
M3	8.5	11.5	14.0	17.5	19.5	23.5

Mandible	Cusp initiation	Crown ½	Crown complete	Root ½	Root complete	Apex closed
I1	0.4	1.2	4.0	6.5	7.5	8.5
I2	0.8	2.8	4.0	7.0	8.0	8.5
C	0.9	2.8	5.5	9.5	12.0	14.5
Pm1 (P3)	2.0	3.5	5.5	9.5	12.5	14.0
Pm2 (P4)	2.5	5.5	6.5	11.0	13.0	14.0
M1	0.4	1.9	3.5	6.5	9.0	10.5
M2	2.5	6.0	8.0	10.5	14.5	16.5
M3	8.5	12.5	14.0	16.5	19.5	23.5
